# *FoxO1* Mediated by H3K27me3 Inhibits Porcine Follicular Development by Regulating the Transcription of *CYP1A1*

**DOI:** 10.3390/ani14233514

**Published:** 2024-12-05

**Authors:** Zhi Zhou, Yuanyuan Lv, Liying Li, Xiaolong Yuan, Xiaofeng Zhou, Jiaqi Li

**Affiliations:** State Key Laboratory of Swine and Poultry Breeding Industry, National Engineering Research Center for Breeding Swine Industry, Guangdong Provincial Key Laboratory of Agro-Animal Genomics and Molecular Breeding, College of Animal Science, South China Agricultural University, Guangzhou 510642, China; 13422047701@163.com (Z.Z.); yuanyuanlv85@163.com (Y.L.); leeleeying@163.com (L.L.); yxl@scau.edu.cn (X.Y.)

**Keywords:** H3K27me3, *FoxO1*, *CYP1A1*, follicular development, porcine

## Abstract

It is well known that the function of granulosa cells (GCs) is closely related to follicular development, and *FoxO1* and histone methylation have been implicated in follicular development. However, the specific mechanisms by which *FoxO1* and histone methylation regulate follicular development are still largely unknown. To explore the specific mechanism of *FoxO1* in regulating follicular development, in this study, we showed that the expression of *FoxO1* in immature ovaries and small follicles was significantly higher than in mature ovaries and large follicles of sows, respectively. *FoxO1* was found to inhibit the secretion of testosterone and proliferation of porcine GCs and promote the secretion of progesterone and apoptosis of porcine GCs. Furthermore, H3K27me3, as a transcriptional inhibitor, can inhibit the transcription of *FoxO1*. *FoxO1* could promote the transcription of *CYP1A1*, and *CYP1A1* was found to inhibit the proliferation and facilitate the ferroptosis of porcine GCs. Collectively, our results revealed that the H3K27me3-*FoxO1*-*CYP1A1* pathway might participate in follicular development, and these findings could provide potential targets for improving follicular development in sows.

## Simple Summary

Follicular development is closely related to fertility. In this study, we aimed to explore the specific mechanism of *FoxO1* in regulating follicular development. Our findings revealed that the H3K27me3-FoxO1-CYP1A1 pathway might be a potential target for improving follicular development in sows.

## 1. Introduction

In mammals, the ovary is an essential reproductive organ, and the follicles are the fundamental and functional unit in the ovaries [1]. The ovaries serve as the final effector organ of the hypothalamic–pituitary–ovarian axis, the main function of which is to nurture mature follicles and ovulate oocytes. The follicle grows from the primordial follicle to the primary, secondary, and tertiary follicles [2], but more than 99% of the primordial follicles are atretic and degenerate at different stages of development [3], and only a few follicles mature and ovulate through recruitment, selection, and dominance processes [4]. The ovarian follicle consists of the oocytes, granulosa cells (GCs), and theca cells, and GCs are closely associated with follicular development and atresia. It has been shown that apoptosis of GCs is a direct inducer of follicular atresia [5]. GCs in atretic follicles exhibit several changes, such as nuclear chromatin fixation, activation of Caspase family enzymes, and DNA breaks between ribosomes [6,7,8], leading to massive apoptosis of GCs. Zhou et al. showed that DNA hypomethylation promoted the expression of *RSPO2* during follicular development, yet suppression of *RSPO2* might inhibit porcine follicular development by promoting the apoptosis of GCs [9]. However, the mechanisms by which proliferation or apoptosis of GCs affect follicular development are unclear.

*FoxO1* is a major member of the FoxO subfamily and exerts a wide range of biological effects in many physiological processes such as cell growth, proliferation, apoptosis, cell cycle arrest, oxidative stress tolerance, and metabolism [10]. *FoxO1* regulates ovarian function mainly through the regulation of GCs apoptosis and is essential for normal reproduction in animals. Studies have shown that *FoxO1* may regulate follicular development and atresia in mammals such as mice, pigs, and cattle [11]. H3K27me3 is a repressive histone modification whose main role is to repress the transcription of target genes [12]. H3K27me3 and its regulatory enzymes play important roles in various biological processes such as growth and development, metabolic regulation, and immune response in animals by repressing the transcription of genes [13]. It has been shown that H3K27me3 inhibits the expression of progesterone receptor and leads to progesterone resistance phenotype in endometrial cancer [14]. Genome-wide scanning of H3K27me3 modification sites in the ovary using ChIP-Seq technology revealed H3K27me3 modification sites on the DNA sequence of *FoxO1*, so we hypothesized that H3K27me3 may bind to the promoter of *FoxO1* to repress its transcription [15]. Okada et al. found that human chorionic gonadotropin might promote the expression of *CYP11A1* by reducing H3K27me3 levels at the *CYP11A1* promoter in rats [16]. *CYP1A1* is a member of the cytochrome P450 superfamily, which is an important metabolic enzyme. Currently, the body mainly achieves the regulation of *CYP1A1* by regulating the AHR signaling pathway. It was found that *CYP1A1* was detected in the GCs growing stage follicles, and *CYP1A1* may affect the growth and survival of ovarian GCs [17].

In this study, to confirm whether the *FoxO1* gene is involved in follicular development, we analyzed the expression of *FoxO1* in ovarian tissues and follicles. To further explore the specific mechanism of *FoxO1* in follicular development, the regulation of *FoxO1* by H3K27me3 and the effect of *FoxO1* on GCs function were examined. In addition, we explored the role of *FoxO1* in regulating the transcriptional expression of the target gene *CYP1A1*, which in turn affected the proliferation and apoptosis of ovarian GCs. These works laid the foundation for further elucidation of the mechanisms by which the H3K27me3-*FoxO1*-*CYP1A1* pathway affects follicular development in sows.

## 2. Materials and Methods

### 2.1. Animals and Sample Preparation

The immature ovaries were collected from three Landrace × Yorkshire crossbred gilts (age = 162 ± 3 days, weight = 81.38 ± 2.40 kg, pre-pubertal), and the mature ovaries were collected from three Landrace × Yorkshire crossbred gilts (age = 212 ± 14 days, weight = 110 ± 2 kg, in-pubertal). The entire portion of three immature or mature pig ovaries were used for *FoxO1* mRNA measurement. These 15 tissues were collected from three Landrace × Yorkshire crossbred gilts (age = 212 ± 14 days, weight = 110 ± 2 kg, in-pubertal). Small (<3 mm) and large follicles (>5 mm) were collected from prepubertal sows at a local slaughterhouse.

### 2.2. Porcine Granulosa Cells Culture and Transfection

Ovaries of prepubertal sows collected from local slaughterhouses were immersed in phosphate-buffered saline (PBS) (Invitgen, Shanghai, China) containing penicillin (100 IU/mL) and streptomycin (100 μg/mL) and transported to the laboratory, where ovaries continued to be rinsed several times with phosphate-buffered saline containing penicillin and streptomycin. GCs were isolated from ovarian follicles that were pooled. Subsequently, GCs were aspirated by inserting a syringe into a 3–5 mm medium follicle, centrifuging and discarding the supernatant, and washing twice with PBS. The cells were then inoculated into culture flasks containing 10% fetal bovine serum and incubated at 37 °C with a CO_2_ concentration of 5%. After 24 h, the medium was changed to remove non-adherent cells and incubation was continued in an incubator, with culture medium changes every 48 h. When the cell density reached 70% to 80%, transfection experiments were performed with reference to the Lipofectamine^®^ 3000 Transfection Kit (Thermo Scientific, Waltham, MA, USA) and incubated in the incubator.

### 2.3. 5-Ethynyl-2′-Deoxyuridine (EdU) Assay

Cell proliferation was analyzed with the CELL-LIGHTTM EDU Apollo 567 in vitro kit (Guangdong, RiboBio, Guangzhou, China). GCs were cultured on 48-well plates for 24 h. The appropriate amount of 50 μM EdU medium was prepared by diluting EdU solution with cell complete medium at a ratio of 1000:1, and 100 μL was added to each well and incubated for 2 h. The medium was discarded, and the cells were washed twice with PBS and then incubated for 30 min with 80% acetone. GCs were washed 2 times with PBS, and then 0.5% Triton X-100 was added to the PBS for 10 min. We added 1 × Apollo and incubated for 30 min at room temperature, discarded the staining reaction solution. The nuclei were washed twice with 0.5% Triton X-100 in PBS and stained with Hoechst 33342 reaction solution, and then incubation continued for 30 min at room temperature, protected from light. Finally, three fields of view were randomly selected from each well, and the number of granulosa cells was counted under an inverted fluorescence microscope for statistical analysis.

### 2.4. Flow Cytometry

Cell cycle distribution was analyzed by flow cytometry. After treating the cells for 24 h, the cells were fixed with 70% ethanol and then further washed with 1% bovine serum albumin and incubated overnight at 4 °C with propidium iodide (PI) staining mixture, protected from light, and the fluorescence of individual cell nuclei was analyzed by flow cytometry (BD FACScan; BD Biosciences, Franklin Lakes, NJ, USA).

Apoptosis rate was analyzed by flow cytometry. Referring to the instructions of the Annexin V-FITC/PI double-stained apoptosis assay kit, GCs were cultured in 6-well plates for 24 h. The collected cells were centrifuged at 1000 rpm for 5 min, supernatant was removed, and cells were washed twice with PBS. Then, 500 μL of 1× Annexin V buffer was added to slowly suspend the cells, and 5 μL of Annexin V-isothiocyanate fluorescein and 10 μL of PI staining solution were added and mixed. The cells were incubated for 15 min at room temperature under dark conditions and then subjected to flow cytometry.

### 2.5. Enzyme Linked Immunosorbent Assay (ELISA)

The synthesis and secretion of steroids in GCs were detected according to the instructions of a porcine enzyme-linked immunosorbent assay (ELISA) kit. Porcine Testosterone Kit (ml002339) and Porcine Progesterone Kit (ml002422) were purchased from Enzyme-linked Biotechnology (Shanghai, China). Different concentrations of 50 μL of reference material were added to the standard well, and the supernatant of the cell culture medium was collected and centrifuged to remove impurities. Then, 10 μL supernatant was added to the sample well containing 40 μL sample diluent and incubated at 37 °C for 30 min to remove the liquid. We added detergent, left to stand for 30 s, removed the liquid, and repeated this step five times. We added 50 μL of chromogenic agent tmb to each, incubated them for 15 min at 37 °C, added termination solution, and determined the optical density at 450 nm.

### 2.6. Plasmid Construction and Dual-Luciferase Reporter Assay

Based on the CDS region sequence information of the porcine *FoxO1* and *CYP1A1* in the NCBI database, BioEdit software 7.0 version was used to analyze the distribution of digestion sites in the CDS region, and then the PCR amplification primers were designed using Primer premier 5.0 and cloned into the expression vector pcDNA3.1. Purification of plasmids was performed by the endotoxin-free plasmid DNA small extraction kit with its instructions. All siRNAs synthesized by Ribo Bio (Guangzhou, China) were transfected into 80% confluent GCs with liposomal^TM^3000 reagent (Thermo Science, Waltham, MA, USA) for 24 h. All luciferase activities were determined using a double luciferase reporter kit (Promega, Madison, WI, USA) and normalized to Renilla luciferase activity.

We constructed the overexpression vector (pcDNA3.1-*FoxO1*) and interference fragment (si-*FoxO1*) of *FoxO1* and verified its transfection efficiency. We chose 300 ng as the optimal transfection concentration for pcDNA3.1-*FoxO1* and 50 nM for si-*FoxO1*. In addition, we also constructed eukaryotic expression vector pcDNA3.1-*CYP1A1* and synthesized interference fragment si-*CYP1A1*, then we transfected them into GCs and verified the overexpression and interference efficiency of *CYP1A1* gene, using 100 ng as the optimal transfection concentration for pcDNA3.1-*CYP1A1* and 100 nM for si-*CYP1A1*.

The promoter −1976/+210 bp, −1407/+210 bp, −1262/+210 bp, and −802/+210 bp regions of *CYP1A1* were amplified from the GCs genome. Amplified samples were cloned into the pGL3 vector to obtain pGL3-C1, pGL3-C2, pGL3-C3, and pGL3-C4 recombinant vectors. The vectors were transfected into GCs and assayed for luciferase reporter gene activity.

### 2.7. Real-Time PCR

Total RNA was extracted from porcine ovarian GCs using TRIzol reagent (Takara, Tokyo, Japan), and RNA concentration was determined with NanoDrop (Thermo Fisher Science, Waltham, MA, USA). Reverse transcription mRNAs were obtained using RT Kit (Thermo Science, Waltham, MA, USA), and 2× Maxima SYBR Green/ROX qPCR Master Mix (Thermo Science, Waltham, MA, USA) was used to quantify the relative expression levels of mRNAs in the CFX96 Touch Real-time PCR System (Bio-Rad, Berkeley, CA, USA). Using GAPDH as the internal reference gene, the relative expression level of the gene was calculated by the 2^−ΔΔct^ method. The primer sequences used in pigs are listed in Table 1.

### 2.8. Chromatin Immunoprecipitation Assay

Chromatin immunoprecipitation (ChIP) was detected with the ChIP kit (ChIP-IT High Sensitivity, Active Motif, Carlsbad, CA, USA). Porcine GCs were crosslinked with 1% formaldehyde at room temperature and then washed with PBS, and cells were collected. The lysed cells were centrifuged, and the supernatant was preserved. Then, 5 μL of lysed sample was used for analysis as Input, and further IP trials were performed using 45 μL. In addition, 10 μL of equal amounts of H3K27me3/FoxO1 antibody (FoxO1), 10 μL of positive RNA polymerase II antibody, and 10 μL of negative immunoglobulin were added to each IP solution, which was then incubated overnight. Protein A agarose beads (10 μL) were washed three times with lysis buffer, slowly mixed with IP solution in a rotary mixer for 2 h, washed with eluent, and centrifuged at 4000 rpm at 4 °C for 5 min, and eluate buffer was added to collect the binding DNA. The DNA was purified with a silica gel column and dissolved in elution buffer. Primers for polymerase chain reaction are listed in Table 2. Each group of qPCR has three replicates.

### 2.9. Fe^2+^ Level Assay

Fe^2+^ level assay was performed by using the Cell Total Iron Colorimetric Assay Kit (Elabscience, Wuhan, China). The supernatants of lysed cells were incubated with iron reductase reagent for 40 min. The optical density at 593 nm was measured.

### 2.10. Statistical Analysis

All the experiments were repeated three times, and the data are expressed as mean ± standard deviation (SD). When comparing the two groups of data, students’ *t*-test was carried out with GraphPad Prism7.0 software. In addition, * means *p* < 0.05, ** means *p* < 0.01, and *** means *p* < 0.001.

## 3. Results

### 3.1. FoxO1 Inhibits Follicular Development in Ovaries of Pigs

**Figure 1:**
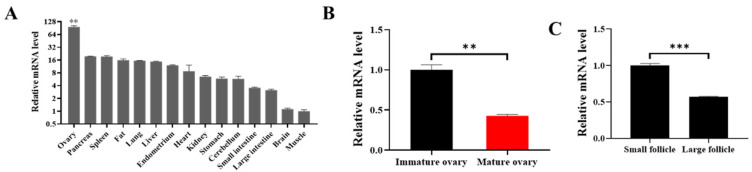
*FoxO1* inhibits the development of follicles. (**A**) The mRNA expressions of *FoxO1* in 15 tissues. (**B**) The mRNA expression of *FoxO1* in immature and mature ovaries. (**C**) The mRNA level of *FoxO1* in small and large follicles. *N* = 3, ** *p* < 0.01, *** *p* < 0.001.

*FoxO1* is differently expressed in 15 porcine tissues, including spleen, fat, and muscle, and the expression of *FoxO1* in ovaries is the highest (Figure 1A). To further investigate the role of *FoxO1* in follicles, we found that the mRNA expression of *FoxO1* in immature ovaries was significantly higher than that in mature ovaries (Figure 1B), and the mRNA expression of *FoxO1* in small follicles (<3 mm) was significantly higher than that in large follicles (>5 mm) (Figure 1C). These results suggest that *FoxO1* might delay the development of follicles.

### 3.2. FoxO1 Promotes the Secretion of Progesterone and Inhibits the Growth of GCs

**Figure 2:**
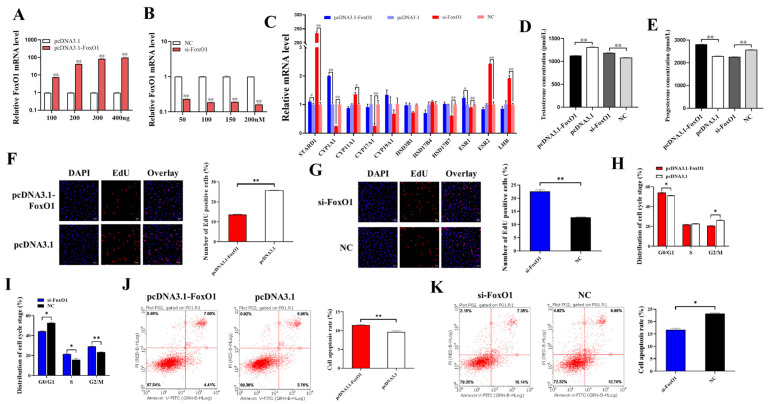
The effects of *FoxO1* on cellular function and hormone levels. (**A**) The mRNA expression of *FoxO1* after transfection of pcDNA 3.1-*FoxO1*. (**B**) The mRNA expression of *FoxO1* after transfection of si-*FoxO1*. (**C**) Effect of *FoxO1* on mRNA expression of genes related to steroid hormone synthesis. (**D**,**E**) Effect of *FoxO1* on testosterone and progesterone secretion in GCs. (**F**,**G**) Effects of overexpression of or interference with *FoxO1* on GCs proliferation. (**H**,**I**) Effects of overexpression of or interference with *FoxO1* on GCs cycle progression. (**J**,**K**) Effects of overexpression of or interference with *FoxO1* on apoptosis of GCs. *N* = 3, * *p* < 0.05, ** *p* < 0.01.

Ovarian secretion of steroid hormones regulates follicular growth, atresia, and GCs apoptosis. We first examined the transfection efficiency of pcDNA3.1-*FoxO1* and si-*FoxO1* (Figure 2A,B). The mRNA level of *FoxO1* was significantly increased and decreased in GCs transfected with pcDNA3.1-*FoxO1* and si-*FoxO1*, respectively, and the overexpression and inhibition effect of *FoxO1* increased with the increase in vector concentration. *FoxO1* upregulated the mRNA expression of *STARD1* (*p* < 0.05), *CYP1A1* (*p* < 0.01), *CYP19A1*, *HSD17B7*, and *ESR1* (*p* < 0.05) and downregulated the mRNA expression of *CYP11A1* (*p* < 0.05), CYP17A1 (*p* < 0.01), *HSD3B1*, *HSD17B4*, *ESR2* (*p* < 0.01), and *LHR* (*p* < 0.05) (Figure 2C). In addition, we measured testosterone and progesterone concentrations in GCs culture supernatant and found that *FoxO1* significantly inhibited the synthesis and secretion of testosterone (Figure 2D) but significantly promoted the synthesis and secretion of progesterone (Figure 2E).

In addition, GCs were transfected with pcDNA3.1-*FoxO1* or si-*FoxO1* to assess cell proliferation, cell cycle progression, and apoptosis. The results of cell proliferation showed that after overexpression of *FoxO1*, the proliferation rate of GCs was significantly lower than that of the control group pcDNA3.1 (Figure 2F), and after interference with *FoxO1*, the proliferation rate of GCs was significantly increased compared to the control NC (Figure 2G), which indicated that *FoxO1* was able to inhibit the proliferation of GCs. Figure 2H showed that *FoxO1* blocked cells in the G0/G1 phase, while the percentage of cells in the G2/M phase was significantly lower than in the control group. After interfering with *FoxO1*, the proportion of cells blocked in the G0/G1 phase was significantly lower than that in control group NC, while the proportion of cells in the S and G2/M phases was significantly increased (Figure 2I). This suggests that *FoxO1* can inhibit the division of GCs and delay the process of cell cycle. The results of apoptosis showed that the apoptosis rate of the pcDNA3.1-*FoxO1* group was significantly higher than that of the pcDNA3.1 group (Figure 2J), while that of the si-*FoxO1* group was significantly lower than that of the NC group (Figure 2K), suggesting that *FoxO1* could promote the apoptosis of GCs.

### 3.3. H3K27me3 Inhibits the Transcription of FoxO1

**Figure 3:**
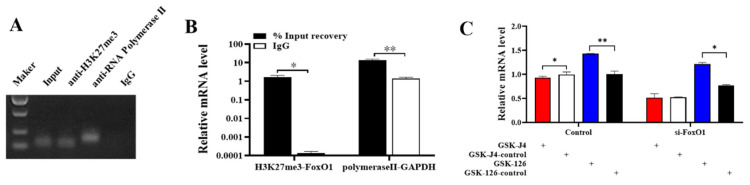
H3K27me3 regulates the transcription of *FoxO1*. (**A**,**B**) ChIP-qPCR validates the combination of H3K27me3 and *FoxO1* promoter region, based on semi-quantitative PCR and qPCR results. (**C**) The effect of H3K27me3 on *FoxO1* mRNA expression. *N* = 3, * *p* < 0.05, ** *p* < 0.01.

ChIP-qPCR results showed that H3K27me3 was bound to the promoter region of *FoxO1* (Figure 3A), and the enrichment abundance of *FoxO1* in H3K27me3 was significantly higher than that of the negative control IgG (Figure 3B). We then treated GCs with H3K27me3 agonist (GSK-J4) and an inhibitor (GSK-126) to verify their effects on *FoxO1*. Results showed that the upregulation of H3K27me3 by GSK-J4 significantly inhibited the mRNA expression of *FoxO1*, and GSK-126 significantly promoted the mRNA expression of *FoxO1*. (Figure 3C). Furthermore, when co-transfecting GCs with GSK-J4 or GSK-126 and si-*FoxO1*, upregulation of H3K27me3 inhibited *FoxO1* mRNA expression more than cells not transfected with si-*FoxO1* (Figure 3C). This suggests that H3K27me3, as a transcriptional inhibitor, can inhibit the transcription of *FoxO1* in porcine ovarian GCs.

### 3.4. FoxO1 Regulates the Transcription of CYP1A1

**Figure 4:**
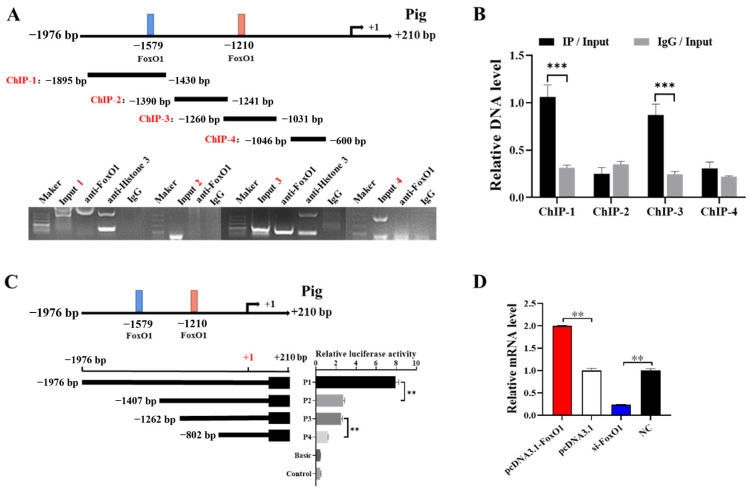
*FoxO1* regulates the transcription of *CYP1A1*. (**A**,**B**) Predicted binding site of *FoxO1* on the *CYP1A1* promoter, which is distinguished into four segments: ChIP-1, ChIP-2, ChIP-3, and ChIP-4. ChIP verified the binding of *FoxO1* and *CYP1A1* promoter region, based on PCR results and enrichment. (**C**) Relative luciferase activity of *CYP1A1* promoter after truncation of *FoxO1* predicted binding site 5′. (**D**) Effect of *FoxO1* on *CYP1A1* mRNA expression in GCs. *N* = 3, ** *p* < 0.01, *** *p* < 0.001.

In this study, bioinformatic predictions and analyses were performed using databases dedicated to transcription factors and their binding sites, such as TFBIND, TRANSFAC [18], and JASPAR [19]. The analysis revealed two potential binding sites for the *FoxO1* transcription factor (−1579/−1569 and −1210/−1200) in the promoter region of *CYP1A1*. For this purpose, we segmented the promoter region of *CYP1A1* and labeled ChIP-1 (−1895/−1430 bp), ChIP-2 (−1390/−1241 bp), ChIP-3 (−1260/−1031 bp), and ChIP-4 (−1046/−600 bp). The binding site of *FoxO1* in the *CYP1A1* promoter region was detected by ChIP, and the direct outcome revealed that *FoxO1* had electrophoretic bands in both ChIP-1 and ChIP-3 regions and that the positive control (Anti-Histone 3) had bands while the negative control (IgG) had almost no bands (Figure 4A). Analysis of the PCR results revealed that *FoxO1* bound more significantly in the ChIP-1 region and that the IP group bands were brighter than the Input group bands (Figure 4B). Subsequently, fluorescence activity analysis was carried out on the promoter region of *CYP1A1*, *CYP1A1* was truncated into P1 (−1976/+210 bp), P2 (−1407/+210 bp), P3 (−1262/+210 bp), and P4 (−802/+210 bp), and significant differences in fluorescence activity were found in the P1-P1 and P3-P4 regions (Figure 4C). To further investigate the relationship between *FoxO1* and *CYP1A1*, pcDNA3.1-*FoxO1* and si-*FoxO1* were respectively transfected into GCs. Compared with the control group pcDNA3.1, the overexpression of *FoxO1* significantly promoted the expression of *CYP1A1* mRNA in GCs. Moreover, compared with the control group NC, the expression of *CYP1A1* mRNA was significantly inhibited after interfering with *FoxO1* (Figure 4D). This suggests that *FoxO1* can target and promote the expression of *CYP1A1*.

### 3.5. CYP1A1 Inhibits GCs Proliferation

**Figure 5:**
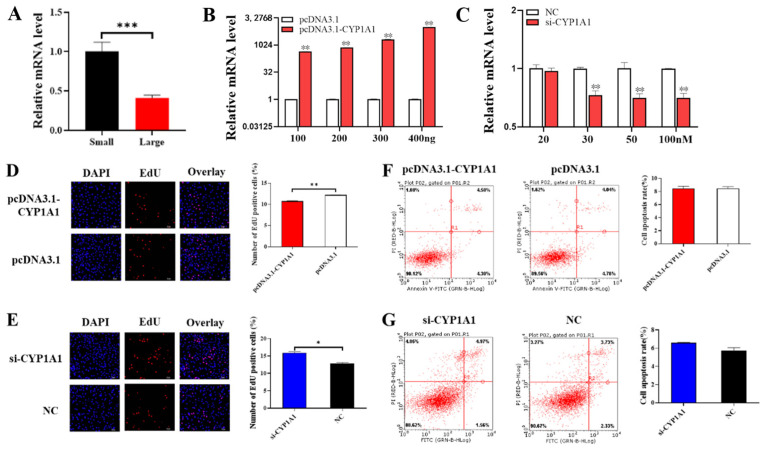
*CYP1A1* inhibits GCs proliferation. (**A**) The mRNA level of *CYP1A1* in small and large follicles. (**B**,**C**) The mRNA expression of *FoxO1* after transfection of pcDNA3.1-*FoxO1* or si-*FoxO1*. (**D**,**E**) The effect of overexpression of or interference with *CYP1A1* on cell proliferation. (**F**,**G**) The effect of overexpression of or interference with *CYP1A1* on cells apoptosis. *N* = 3, * *p* < 0.05, ** *p* < 0.01, *** *p* < 0.001.

In order to explore the effect of *CYP1A1* on the function of ovarian GCs, we first detected the expression of *CYP1A1* in small follicles and large follicles and found that the mRNA level of *CYP1A1* was higher in small follicles and significantly downregulated with follicular development (Figure 5A). In addition, we constructed eukaryotic expression vector pcDNA3.1-*CYP1A1* and synthesized interference fragment si-*CYP1A1*, then transfected them into GCs so as to detect the changes of proliferation and apoptosis of GCs. We verified the overexpression and interference efficiency of *CYP1A1*, using 100 ng as the optimal transfection concentration for pcDNA3.1-*CYP1A1* and 100 nM for si-*CYP1A1* (Figure 5B,C). PcDNA3.1-*CYP1A1* and si-*CYP1A1* were transfected into GCs to detect cell proliferation and apoptosis, respectively. Figure 5D–F showed that *CYP1A1* was able to inhibit the proliferation of GCs but might have no effect on the apoptosis of GCs.

### 3.6. CYP1A1 Facilitates GCs’ Ferroptosis

**Figure 6:**
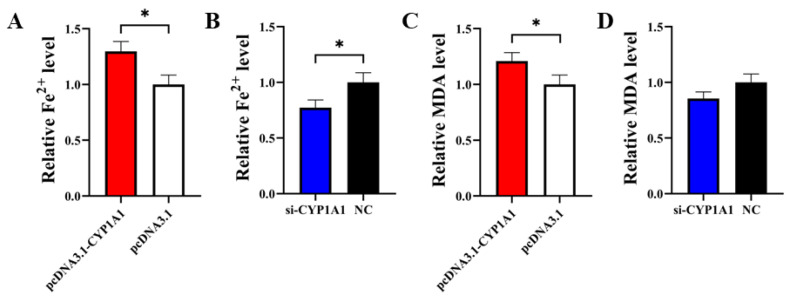
*CYP1A1* inhibits GCs’ proliferation. (**A**,**B**) The effect of overexpression or interference with *CYP1A1* on the Fe^2+^ level in GCs. (**C**,**D**) The effect of overexpression or interference with *CYP1A1* on the MDA level in GCs. *N* = 3, * *p* < 0.05.

PcDNA3.1-*CYP1A1* and si-*CYP1A1* were respectively transfected into GCs to detect the role of *CYP1A1* on cell ferroptosis. The result of the Fe^2+^ level assay showed that pcDNA3.1-*CYP1A1* significantly elevated the Fe^2+^ level in GCs and si-*CYP1A1* exhibited the opposite effect (Figure 6A,B). In the subsequent MDA assay, pcDNA3.1-CYP1A1 significantly elevated the MDA level in GCs but si-*CYP1A1* exhibited an insignificant effect (Figure 6C,D).

## 4. Discussion

Many endocrine, paracrine, and autocrine factors are capable of regulating follicular growth and atresia in sows. Studies have shown that *FoxO1* also has a potential regulatory function on follicle development. FSH causes inactivation of FoxO1 protein phosphorylation through the PKB/AKT pathway and transfers from the nucleus to the cytoplasm, thereby maintaining follicle survival [20]. *Foxo1* was found to be located in all GCs of healthy growing follicles, including primary, secondary, multilayer, and antral follicles. In addition, the initial mechanism of follicular atresia may involve *FoxO1*, whose expression disappears after follicular morphological degradation [21]. In this study, we found that *FoxO1* mRNA was expressed at different levels in 15 tissues, including ovary, pancreas, spleen, and adipose, and the expression of *FoxO1* was highest in ovarian tissue (Figure 1A), indicating that *FoxO1* is essential for the maintenance of normal ovarian function. Before the follicle develops and matures to ovulation, most of the follicles undergo atresia and degenerate. In addition, it has been shown that more oocytes from medium and large follicles have the ability to mature than oocytes from small follicles [22]. In the present study, we also found that the mRNA level of *FoxO1* was significantly higher in immature ovaries than mature ovaries (Figure 1B), and the mRNA level of *FoxO1* was higher in small follicles and significantly downregulated in large follicles (Figure 1C); therefore, we speculate that *FoxO1* may regulate follicular development during ovarian development in sows.

Ovarian secretion of steroid hormones regulates follicular growth, atresia, and the apoptosis of GCs. *FoxO1* plays a key role in regulating lipid and sterol biosynthesis in GCs, thereby preventing elevated steroidogenesis during the early stages of follicular development [23]. In addition, *FoxO1* inhibits the synthesis and secretion of steroid hormones in mouse GCs, which induces apoptosis and leads to follicular atresia. Studies have shown that estrogen activates primordial follicles and promotes GCs proliferation as well as follicular development. Androgens are the substrate for estrogen synthesis, and GCs can use androgens to synthesize estrogen required by the body. On the contrary, progesterone inhibits GCs proliferation and follicle development and promotes the apoptosis of GCs, which further causes follicular atresia. In atretic follicles, the progesterone level is elevated, as is the ratio of progesterone to estrogen [24]. In this study, our experimental data showed that *FoxO1* inhibited the synthesis and secretion of testosterone (Figure 2D) and promoted the synthesis and secretion of progesterone (Figure 2E), which is in general agreement with the results of previous studies. Therefore, we hypothesized that FoxO1 may affect the synthesis and secretion of steroid hormones such as estradiol, testosterone, and progesterone in GCs by regulating the mRNA expression of genes related to the steroid hormone synthesis pathway. These results suggest that *FoxO1* can regulate ovarian steroid hormone secretion, which in turn affects follicular development and induces follicular atresia. We also examined the mRNA expression of genes related to steroid hormone synthesis by qRT-PCR and found that *FoxO1* could indeed affect the synthesis and secretion of steroid hormones.

The initiation of primordial follicle growth is mainly dependent on the proliferation and differentiation of GCs [25], while *FoxO1* has been shown to play a key role in the physiological processes of cell proliferation, apoptosis, and cycling. *FoxO1* promotes apoptosis, slows the cell cycle, and blocks cells in the G1 phase [26]. In cancer, *FoxO1* can regulate many target genes, including those involved in apoptosis, cell cycle arrest, and immune regulation [27]. *FoxO1* may achieve its regulation of follicular development by regulating the proliferation and apoptosis of GCs. It was found that *FoxO1* inhibited GCs proliferation in mice, which induced the apoptosis of GCs and follicular atresia [28]. The Periplaneta Americana peptide (PAP) inhibited hydrogen peroxide-induced apoptosis in porcine ovarian GCs by regulating the expression of *FoxO1* [29]. In this study, we transfected pcDNA3.1-*FoxO1* and si-*FoxO1* in GCs to detect changes in the cycle progression, proliferation, and apoptosis of GCs. We found that after overexpression of *FoxO1* in GCs, the cell proliferation rate decreased (Figure 2F), the apoptosis rate increased (Figure 2J), and the percentage of cells blocked in G0/G1 phase increased significantly (Figure 2H), while after interference with *FoxO1*, the cell proliferation rate increased (Figure 2G), the apoptosis rate decreased (Figure 2K), and the percentage of cells blocked in G0/G1 phase decreased noticeably (Figure 2I). This suggests that *FoxO1* plays a role in inhibiting proliferation, promoting apoptosis, and delaying cell cycle progression in porcine ovarian GCs. In our study, GCs were collected from hundreds of 3–5mm follicles for stability, and further studies are needed to confirm the role of *FoxO1* in GCs derived from follicles of other sizes.

H3K27me3 is a chromatin repressor marker that has a critical role in mediating gene silencing. Our previous study revealed that H3K27me3 inhibits the transcription of *RUNX1* during the development and maturation of antral follicles [30]. *RUNX1* acts as an activator of *FSHR*, *CPY11A1*, and *CYP19A1*, promotes the production of androgens, estrogens, and prostaglandins, decreases progesterone levels, and thus affects the proliferation and apoptosis of porcine ovarian GCs. Our group examined the expression of H3K27me3 in follicles of different sizes in the early stage and found that the protein level of H3K27me3 was significantly lower in large follicles than in medium follicles. We also found that the expression of *FoxO1* was higher in small follicles but significantly downregulated in large follicles (Figure 1C), so we hypothesized that H3K27me3 might bind to the promoter sequence of *FoxO1* to repress *FoxO1* transcription. We performed ChIP-qPCR experiments after predictive analysis by a bioinformatics website and verified that H3K27me3 binds to the *FoxO1* promoter to regulate *FoxO1* transcription. In addition, Su et al. found that EZH2 represses *FoxO1* transcription by increasing H3K27me3 on its promoter [31]. Navik et al. examined the enrichment of H3K27me3 and H3K4me3 in the promoter region of *FoxO1* using ChIP and found that H3K27me3 is involved in regulating the expression of *FoxO1* [32]. These experimental results also corroborate the predictions and conclusions of this study.

The modified enzymes of H3K27me3 include a methyltransferase, EZH2, and two de-methyltransferases, KDM6A and KDM6B. GSK-J4 is a dual inhibitor of the de-methyltransferases KDM6A and KDM6B and can act as an agonist of H3K27me3. GSK-126 inhibits the methyltransferase EZH2 and therefore acts as an inhibitor of H3K27me3. In this study, we treated GCs with agonist GSK-J4 and inhibitor GSK-126 and examined the expression of *FoxO1* after 48 h. We found that GSK-J4 significantly inhibited the mRNA expression of *FoxO1*, and GSK-126 significantly promoted the mRNA expression of *FoxO1* (Figure 3C). Meanwhile, we transfected si-*FoxO1* in GCs and then treated the cells with agonist GSK-J4 and inhibitor GSK-126 and found that H3K27me3 inhibited *FoxO1* more strongly (Figure 3C). The above results suggest that H3K27me3 can bind to the promoter region of *FoxO1* to repress the transcription of *FoxO1*.

Specific transcription factors regulate gene transcription by binding to promoter-specific sites and influencing the binding of certain RNA reverse transcription enzymes as well as auxiliary proteins to the promoter region. When we investigated the relationship between *FoxO1* and steroid hormones, we found that *FoxO1* could significantly affect the expression of CYP1A1, so we speculated whether there was a link between *FoxO1* and *CYP1A1* (Figure 2A). It was shown that knockdown of *CYP1A1* or inactivation of AKT signaling using LY294002 downregulated p-AKT in vitro and vivo experiments, thereby inhibiting cell proliferation [33]. In this study, bioinformatic predictions and analyses using databases dedicated to transcription factors and their binding sites, such as TFBIND, TRANSFAC [18], and JASPAR [19], revealed that the *CYP1A1* promoter region contains binding sites for the *FoxO1* transcription factor. In addition, we detected the expression of *CYP1A1* by qRT-PCR after transfecting pcDNA3.1-*FoxO1* and si-*FoxO1* in GCs and found that overexpression of *FoxO1* significantly promoted *CYP1A1* expression and interference with *FoxO1* significantly inhibited *CYP1A1* expression, so we speculated that *FoxO1* might target the promotion of *CYP1A1* expression. To investigate this further, we investigated the relationship between *FoxO1* and *CYP1A1* and verified *FoxO1* binding to the *CYP1A1* promoter region by ChIP (Figure 4A). We further investigated the role of *CYP1A1* in cell proliferation and apoptosis. We found that overexpression of *CYP1A1* significantly reduced the proliferation rate of GCs, while interference with *CYP1A1* did the contrary, suggesting that *CYP1A1* acts as an inhibitor of cell proliferation in GCs. However, we also found that *CYP1A1* may have no significant effect on apoptosis of GCs. Nonetheless, it has also been shown that upregulation of *CYP1A1* induces apoptosis induction and oxidative stress [34]. DIM (3,3′-Diindolylmethane) activates the AHR pathway and induces *CYP1A1* expression in human gastric carcinogenesis cells, thereby inhibiting cell proliferation, delaying cell cycle progression, and inducing apoptosis in vitro [35].

## 5. Conclusions

In conclusion, H3K27me3 inhibits the transcription of *FoxO1*, and *FoxO1* promotes the apoptosis and progesterone secretion of GCs and inhibits the cycle progression, proliferation, and testosterone secretion of GCs. Its potential target gene, *CYP1A1*, can also significantly inhibit the proliferation of GCs. In other words, *FoxO1* may block the growth and development of ovarian follicles by promoting the transcription of *CYP1A1* and inhibiting the proliferation of porcine ovarian GCs. These studies provide a foundation for further elucidation of the mechanism by which the H3K27me3-*FoxO1*-*CYP1A1* pathway affects follicular development.

## Figures and Tables

**Table 1:** Primers used for PCR.

Gene	Sequence (5′-3′)	Product
*FoxO1*	F: GACAGACTGGGCAGAGTAGAA R: AGCAACGATGACTTTGATAAC	182 bp
*CYP1A1*	F: TATCCTCCGTTACCTGCCCA R: TGCGCCCCTTCTCAAAGATT	119 bp
*STARD1*	F: GCTTTTCCACTCTAGGGCGA R: AAGCTCCTGGCTGGATGAAC	184 bp
*CYP11A1*	F: CGCTCAGTCCTGGTCAAAGG R: TTCCAAGTTGCCGAGCTTCT	267 bp
*CYP17A1*	F: AAGCCAAGACGAACGCAGAAAG R: TAGATGGGGCACGATTGAAACC	228 bp
*CYP19A1*	F: TTCCTTGGCTGTACAGAAAGTATGA R: GGTGTCTGGTGCTGCAATTAG	221 bp
*HSD3B1*	F: ATCGTCCACTTGTTGCTGGA R: TGCTCTGGAGCTTAGAAAATTCC	103 bp
*HSD17B4*	F: GAACTTCTACGGGCGTGT R: CCCTCAGAATTCCAGCATTGTT	299 bp
*HSD17B7*	F: TGGACTTCACCTGTGCTTGG R: TGCTGACATCCACTTGCACA	116 bp
*ESR1*	F: ATGGCCATGGAATCTGCCAA R: CCCCTTTCATCATGCCCACT	241 bp
*ESR2*	F: GCCGACAAGGAACTGGTACA R: GAGCAAAGATGAGCTTGCCG	169 bp
*LHR*	F: ACATAACCACCGTACCAGCA R: GGAAGGCGTCATTGTGCATC	177 bp
*GAPDH*	F: TCGGAGTGAACGGATTTG R: TCACCCCATTTGATGTTGG	250 bp

**Table 2:** Segment primers used for *CYP1A1* promoter reporter construction.

Gene	Sequence (5′-3′)	Product
ChIP-1	F: CTCCCTCCCTCAAGGACCACT	466 bp
	R: CTGATGGCCCAGGTCAGAAAA	
ChIP-2	F: GTTGGGGACACGTTGAGCTA	150 bp
	R: ATGCCTATAGCTGGACACGC	
ChIP-3	F: GCGTGTCCAGCTATAGGCAT	230 bp
	R: CTCGGTCCGATACAGTCACG	
ChIP-4	F: ACTGTATCGGACCGAGCCT	477 bp
	R: TAGCAGAAGTCTGTGCTCCC	

## Data Availability

The original contributions presented in the study are included in the article, further inquiries can be directed to the corresponding author.
